# Molecular species fingerprinting and quantitative analysis of saffron (*Crocus sativus* L.) for quality control by MALDI mass spectrometry†

**DOI:** 10.1039/c8ra07484d

**Published:** 2018-10-23

**Authors:** Donatella Aiello, Carlo Siciliano, Fabio Mazzotti, Leonardo Di Donna, Constantinos M. Athanassopoulos, Anna Napoli

**Affiliations:** Department of Chemistry and Chemical Technologies, University of Calabria Italy amc.napoli@unical.it; Department of Pharmacy, Health and Nutritional Sciences, University of Calabria Italy; Department of Chemistry, University of Patras Patras Greece

## Abstract

Herein we describe a rapid, simple, and reliable method for the quantitative analysis and molecular species fingerprinting of saffron (*Crocus sativus* L.) by direct MS and MS/MS analysis. Experimentally, powdered saffron was subjected to a brief treatment with a 0.3% TFA water/acetonitrile solution, and the resulting mixture was directly placed on the MALDI plate for analysis. This approach allowed the detection of the commonly observed crocins C-1–C-6 and flavonols, together with the identification of the unknown highly glycosylated crocins C-7, C-8 and C-9, and carotenoid-derived metabolites. The strategy endorsed the simultaneous detection and characterization of saffron and adulterant markers using crude extracts of the adulterant itself and synthetic sets of adulterated authentic saffron samples. The implementation of the strategy was to measure the amount of an unknown adulterant from the crude extract using curcumin as a non-isotopic isobaric internal standard. The relationship between the saffron and curcumin molar ratios were established with a correlation coefficient of 0.9942. The ANOVA regression model was significant, *F*(1, 72) = 13 595.82, *p* < 0.001, *y* = (0.0116 ± 0.0001)*x* + (−0.1214 ± 0.0086). No matrix effects were observed and good results were obtained with respect to instrumental repeatability (*RSD% < 2%) and LOD (1.1%). The analysis of commercial samples of saffron using the proposed approach showed the suitability of the method for routine analysis (minimal sample preparation and very short measuring time per sample).

## Abstract 2

Herein we describe a rapid, simple, and reliable method for the quantitative analysis and molecular species fingerprinting of saffron (*Crocus sativus* L.) by direct MS and MS/MS analysis.

## Graphical abstract

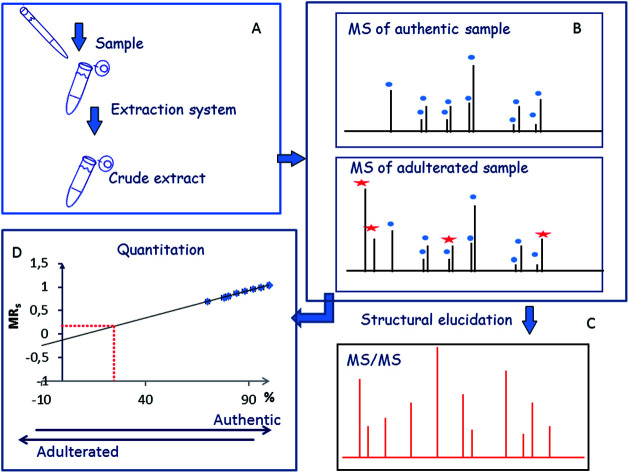

## Introduction

Saffron, which is the red dried stigmas of *Crocus sativus* L., is a member of the Iridaceae family. It is one of the most expensive spices in the world. The major components of saffron are the apocarotenoids *cis*- and *trans*-crocins, picrocrocin and its degradation product the odor-active safranal. It has been suggested that a good quality saffron must contain about 30% crocins, 5 to 15% picrocrocin and more than 0.5% volatile safranal compounds.^1,2^ The coloring strength of saffron depends on the content of crocins.^3,4^ Crocins are glycosyl esters of crocetin (Ct), in which glucose, gentiobiose, neapolitanose, or triglucose (C-1–C-5) are the sugar moieties.^1,5^ Many methods for saffron component analysis have been described in the literature including reverse-phase high-performance liquid chromatography (HPLC) in combination with UV, diode array.^6^ Recently, studies of diffuse reflectance infrared Fourier transform spectroscopy (DRIFTS)^7^ and ^1^H NMR combined with chemometric techniques^8^ have been proposed in evaluating the adulteration of saffron with characteristic adulterants of plant origin. At present, there is a growing tendency to find quick, simple, and powerful tools which enable detection of saffron metabolites for quality and fraud control.^9–12^ Considering that the sensory characteristics (flavor, taste, color) and particular properties (health benefits) of saffron are essentially determined by its chemical content, the application of MS-based chemical component profiling offers significant opportunities to obtain precious detailed information that can be directly correlated to the spice quality. Mass spectrometry is a well-established method for the high-throughput detection and quantitative analysis^13^ of metabolites,^14^ amino acids and their synthetic analogues,^15^ and proteins.^16^ Direct MS analysis of foods or food extracts^17,18^ has been found to be a useful and robust approach for the chemical fingerprinting when rapid classification of food-sample types or rapid screening of food adulteration is wanted. Matrix-assisted laser desorption ionization mass spectrometry (MALDI MS) and tandem mass spectrometry (MS/MS) techniques have seldom been considered for both direct analysis of saffron extracts, and for quantifying adulterants in saffron.^19^ MALDI-TOF-MS has been reported to be a very useful analytical technique for authenticity assessment.^17^ Its advantages include high sensitivity, accuracy, tolerance to buffers and fast data acquisition.^20^ The specificity of MS and MS/MS experiments enable the identification of markers through the elucidation of their molecular structures. Furthermore, MALDI MS system allows quantitative determination of low molecular weight compounds, since special instrument setup and isotopically labelled internal standard are not required.^20^ Chen *et al.* have recently reported a new label-free LDI MS imaging technique, that combine advantage of including both imaging and quantitative analysis.^21^ Limitations of MALDI experiments, such as the strong matrix interference and poor reproducibility of the signal intensity, appear to be solved by averaging multiple analyses of the same spot.^22^

In this work, a simple method MALDI MS based for the fast quality control of saffron was exploited. The strategy developed has different stages: devising of sample preparation protocol to obtain a chemical component profile of saffron by MALDI MS that can be evaluated and matched to retrieve data by simple spectra comparison. MALDI MS component profile followed by MS/MS was employed to detect simultaneously species arising from saffron and adulterating matrix with sufficient abundance for quantitative and MS/MS analysis.

## Experimental

### Chemicals

Solvents (CH_3_CN, and H_2_O, HPLC grade), α-cyano-4-hydroxy-*trans*-cinnamic acid (α-CHCA, pure 99.0%), sinapinic acid (SA, pure 99.0%), and curcumin were purchased from Sigma Aldrich Fluka (Milano, Italy).

### Spice and sample preparation

Samples of saffron spice were directly obtained from producers with a guarantee of their origin and freedom from fraud. The dried *Crocus sativus* L. stigmas were obtained from Cooperative of Saffron, (Krokos Kozanis, Greece). Powdered saffron, *Curcuma longa* (turmeric) and *Calendula officinalis* L. (calendula) were purchased from a local market. *Crocus sativus* L. stigmas, turmeric and calendula crude materials were ground into a fine powder. Aliquots of powdered turmeric, or calendula were used to prepare adulterated saffron samples used in chemical composition experiments. Saffron was homogenized by crushing in a pressure rolling with metallic cylinder. A portion (5 mg) of powdered saffron and adulterated samples were extract with 1 mL of ethanol, methanol/water (50/50: v/v), and 0.3% TFA in H_2_O/CH_3_CN (40 : 60, v/v) solution at room temperature, under magnetic stirring for 2 min. After 2 min centrifugation at 12 000 rpm, the pellet was discarded. A 1 μL portion of each sample was spotted and dried at room temperature. Matrix solution (1 μL), was pipetted onto dried samples. After the crystals had dissolved completely, the spot was dried under a continuous air stream. Matrix solutions were prepared dissolving 20 mg mL^−1^ of SA in 60% CH_3_CN/0.3% TFA in water (v/v).

### Standard solution

A stock standard solution was prepared by dissolving curcumin in CH_3_CN up to a final concentration of 20 mg mL^−1^.

Set of authentic adulterated saffron sample was prepared as follows: a portion (5 mg) of powdered saffron was extracted with 1 mL of the extracting solution under magnetic stirring for 2 min. The extracting solution was prepared for each level of adulteration by adding 0.3% TFA in H_2_O/CH_3_CN (40 : 60, v/v), 1 mM of KCl to the appropriate amount of curcumin solution (20 mg mL^−1^) up to final volume of 1 mL. A 1 μL portion of each sample was spotted and dried at room temperature.

The limit of detection (LOD) and the limit of quantitation (LOQ) were calculated by applying eqn (1) and (2), following the directives of IUPAC and the American Chemical Society's Committee on Environmental Analytical Chemistry. S_LOD_ is the signal at the limit of detection, S_LOQ_ is the signal at the limit of quantitation, S_RB_ is the signal of the blank “authentic saffron samples”, and *σ*_RB_ is the standard deviation.

1 S_LOD_ = S_RB_ + 3*σ*_RB_

2 S_LOQ_ = S_RB_ +10*σ*_RB_

### MALDI-TOF-MS and high energy CID-MS/MS analysis

Each sample was directly spotted three times on a 384-well insert Opt-TOF™ stainless steel MALDI plates (AB SCIEX, Darmstadt, Germany). Mass spectrometric analyses were performed using a 5800 MALDI-TOF-TOF Analyzer (AB SCIEX, Darmstadt, Germany) equipped with an Nd:YLF Laser with *λ* = 345 nm wavelength of <500 ps pulse length and *p* to 1000 Hz repetition rate, in reflectron positive mode with a mass accuracy of 5 ppm. Mass spectra were acquired automatically in the positive reflector mode between 200 and 2000 with a fixed laser intensity. Spectra with signal-to-noise below 200 were discarded automatically by the instrument. The operation parameter was optimized for the mass region of interest. Laser intensity was adjusted manually to avoid detector saturation. At least 4000 laser shots are typically accumulated with a laser pulse rate of 400 Hz in the MS mode, whereas in the MS/MS mode spectra up to 5000 laser shots are acquired and averaged with a pulse rate of 1000 Hz. MS/MS experiments were performed at a collision energy of 1 kV, ambient air is used as collision gas at a pressure of 10^−6^ torr. The potential difference between the source acceleration voltage and the collision cell was set as 1 kV. After acquisition, spectra were handled using Data Explorer version 4.11 (AB Sciex). All data presented in this work are averages of three replicate.

### Nomenclature for crocetin esters

To abbreviate the names of crocetin esters in this paper, they were labeled as follows: the nomenclature were written with a hyphen separating the total number of the glucose moieties at both extremes of the basic molecule (C-n). Then, C-4 would indicate Ct (crocetin), with four hexoses (Hex, glucose residues). The fragment ions were designated according to the nomenclature proposed by Domon and Costello^23^ for glycoconjugates, and referring to the nomenclature of flavonoid fragmentations.^24^

## Results and discussion

### Method design

**Fig. 1:**
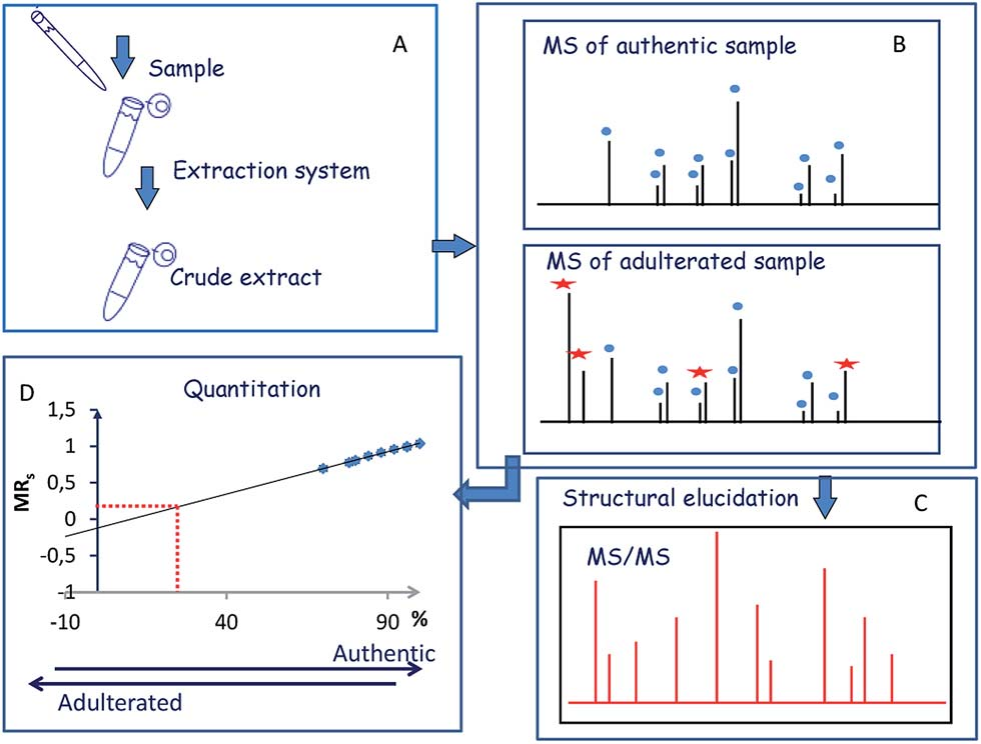
Workflow of the MALDI MS and MS/MS approach.

The first step of the strategy comprised the optimization of extraction procedure to perform the simultaneous detection and characterization of the major components (crocins and picrocrocin) of saffron (Fig. 1A). The second step of the strategy was focused on the simultaneous detection and characterization of saffron and adulterant markers using crude extracts of adulterant itself and synthetic sets of adulterated authentic saffron samples (Fig. 1B and C). Finally, the implementation of the strategy was to measure the amount of an unknown adulterant from the crude extract of fortified authentic saffron samples and saffron sachets purchased from market (Fig. 1D).

### Sample preparation and MALDI MS analysis of saffron

The goal of this study was to use a MALDI-TOF/TOF-MS instrument as a sensitive detection system to perform a fast quality control of saffron in order to detect saffron adulteration without pre- or intermediate purification or desalting procedures. The stigmas of Greek saffron were considered as the model system to elaborate the potential of chemical component profile. The direct MALDI-MS analysis of the sample can be combined with a simple and selective sample preparation preventing analyte losses. The sample preparation can be tailored since saffron marker of authenticity are well established and can be used to optimize the extraction procedure. The hydrophilic properties of crocins suggest possible different accumulation sites inside the chromoplasts.^25^ In cells of saffron mature stigmas the water-soluble crocetin glycosides can be sequestered within the central vacuole which eventually fuses with chromoplast. This led to the hypothesis that the use of a binary solvent system has the potential to disrupt the cell membranes, and should favor the release of crocetin glycosides in aqueous media.^26^ Therefore, direct detection of crocins from saffron using binary solvent system for extraction was planned. The structure of crocins C-1–C-6 from saffron has been elucidated.^27^ Focus was pointed out on ion signals related to C-2 (*m*/*z* 675.27), C-3 (*m*/*z* 837.32), C-4 (*m*/*z* 999.38) and C-5 (*m*/*z* 1161.45). They gave good MS signals enabling the optimization of the extraction procedure (matrix solution preparation, sample/matrix solution ratio). To lyse cells and selectively solubilize metabolites, stigmas were extracted with different solvents such as ethanol, methanol/water, and a mixture 0.3% TFA in H_2_O/CH_3_CN (40 : 60, v/v) (Fig. 1S, ESI†). Among these, the latter solvent system was shown to be most suitable for the solubilization of crocins from in house powered stigmas. Further, 5 mg of powered saffron were extracted using a 0.3% TFA in H_2_O/CH_3_CN (40 : 60, v/v) for two minutes under magnetic stirring, at room temperature. A small aliquot of the crude extract (1 μL) was directly placed on the MALDI sample plate and analyzed. When the extraction time was extended to 5 minutes, no significant changes in the extent of crocins from sample were observed in the recorded MALDI spectra.

### Identification of crocins

The MALDI MS spectrum crude extract showed the specific *m*/*z* spacing patterns (n 162) of crocetin glycosides, providing glycoforms within the 0.5–2.0 kDa mass range. Almost all observable peaks corresponded to C-1–C-9 were unequivocally determined by the accurate mass of each peak (Table 1). Crocins were readily desorbed and ionized by MALDI as cationized species. The MALDI-MS spectrum displayed for all crocins two adduct ions separated by 16 Da ([M + Na]^+^*versus* [M + K]^+^). High energy MS/MS fragmentation of all precursor ions [M + Na]^+^ gave favorable product ions which were appropriate with the systematic structural analysis of crocins.

**Table 1:** Crocins identified in the extract of *Crocus sativus* L. stigmas by high-energy CID-MS/MS

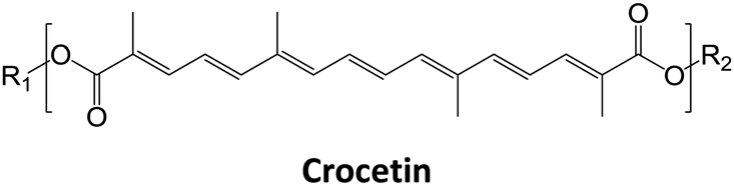					
[C-1 + Na]^+^ MS/MS	R_1_ = Hex; R_2_ = H	[C_26_H_34_NaO_9_]^+^; 513.21			
		[C_20_H_24_NaO_3_]^+^; 335.2	[C_23_H_28_NaO_6_]^+^; 423.2	[C_20_H_24_NaO_4_]^+^; 351.2	[C_9_H_16_NaO_7_]^+^; 259.1
[C-2 + Na]^+^ MS/MS	R_1_ = Hex_2_; R_2_ = H	[C_32_H_44_NaO_14_]^+^; 675.27			
		[C_31_H_44_NaO_12_]^+^; 631.3	[C_29_H_38_NaO_11_]^+^; 585.2	[C_26_H_34_NaO_9_]^+^; 513.2	[C_6_H_10_NaO_5_]^+^; 185.0
		[C_25_H_32_NaO_8_]^+^; 483.2	[C_18_H_30_NaO_12_]^+^; 461.2	[C_12_H_22_NaO_11_]^+^; 365.1	[C_20_H_24_NaO_4_]^+^; 351.2
		[C_10_H_18_NaO_9_]^+^; 305.1	[C_9_H_16_NaO_7_]^+^; 259.1	[C_16_H_20_NaO]^+^; 251.1	
[C-3 + Na]^+^ MS/MS	R_1_ = Hex_2_; R_2_ = Hex	[C_38_H_54_NaO_19_]^+^; 837.32			
		[C_32_H_44_NaO_14_]^+^; 675.1	[C_26_H_34_NaO_9_]^+^; 513.2	[C_18_H_30_NaO_15_]^+^; 509.1	[C_18_H_30_NaO_14_]^+^; 493.1
		[C_12_H_20_NaO_11_]^+^; 363.1	[C_12_H_20_NaO_10_]^+^; 347.1	[C_20_H_24_NaO4]^+^; 351.2	[C_9_H_16_NaO_7_]^+^; 259.1
		[C_6_H_10_NaO_5_]^+^, 185.0			
[C-4 + Na]^+^ MS/MS	R_1_ = Hex_2_; R_2_ = Hex_2_	[C_41_H_58_NaO_21_]^+^; 999.38			
		[C_38_H_54_NaO_19_]^+^; 837.3	[C_32_H_44_NaO_14_]^+^; 675.3	[C_12_H_20_NaO_10_]^+^; 347.1	[C_12_H_20_NaO_11_]^+^; 658.2
		[C_20_H_24_NaO_4_]^+^; 351.2	[C_18_H_30_NaO_15_]^+^; 509.1	[C_24_H_42_NaO_21_]^+^; 689.2	
[C-5 + Na]^+^ MS/MS	R_1_ = Hex_3_; R_2_ = Hex_2_, R_1_ = Hex_4_; R_2_ = Hex_1_	[C_50_H_74_NaO_29_]^+^; 1161.45			
		[C_12_H_20_NaO_10_]^+^; 347.1	[C_44_H_64_NaO_24_]^+^; 999.4	[C_38_H_54_NaO_19_]^+^; 837.3	[C_18_H_30_NaO_15_]^+^; 509.1
		[C_32_H_44_NaO_14_]^+^; 675.3	[C_24_H_40_NaO_20_]^+^; 671.2	[C_26_H_34_NaO_9_]^+^; 513.2	[C_12_H_20_NaO_11_]^+^; 363.1
		[C_38_H_52_NaO_18_]^+^; 819.3	[C_24_H_42_NaO_21_]^+^; 689.2;	[C_26_H_34_NaO_8_]^+^; 497.2	[C_47_H_68_NaO_27_]^+^; 1087.4
		[C_47_H_68_NaO_26_]^+^; 1071.4	[C_40_H_56_NaO_21_]^+^; 895.3	[C_41_H_58_NaO_21_]^+^; 909.3	[C_40_H_58_NaO_20_]^+^; 881.3
		[C_35_H_48_NaO_16_]^+^; 747.3	[C_34_H_46_NaO_16_]^+^; 733.3	[C_21_H_36_NaO_18_]^+^; 599.2	
[C-6 + Na]^+^	R_1_ = Hex_4_; R_2_ = Hex_2_	[C_56_H_84_NaO_34_]^+^;1323.49			
		[C_50_H_74_NaO_29_]^+^; 1161.4	[C_24_H_42_NaO_21_]^+^; 689.2	[C_44_H_64_NaO_24_]^+^; 999.4	[C_38_H_54_NaO_19_]^+^; 837.3
		[C_53_H_78_NaO_31_]^+^; 1233.4			
[C-7 + Na]^+^ MS/MS	R_1_ = Hex_6_; R_2_ = Hex_1_	[C_62_H_94_NaO_39_]^+^1485.54			
		[C_56_H_86_NaO_34_]^+^; 1325.5	[C_50_H_74_NaO_29_]^+^; 1161.4	[C_24_H_42_NaO_21_]^+^; 689.2	[C_36_H_60_NaO_30_]^+^; 995.3
		[C_26_H_34_NaO_9_]^+^; 513.2	[C_30_H_50_NaO_25_]^+^; 833.2	[C_59_H_88_NaO_36_]^+^; 1395.5	[C_26_H_34_NaO_8_]^+^; 497.2
[C-8 + Na]^+^ MS/MS	R_1_ = Hex_6_; R_2_ = Hex_2_	[C_68_H_104_NaO_44_]^+^; 1647.59			
		[C_60_H_90_NaO_37_]^+^; 1425.5	[C_62_H_94_NaO_39_]^+^; 1485.5	[C_24_H_40_NaO_20_]^+^; 671.2	[C_56_H_84_NaO_34_]^+^; 1323.5
		[C_33_H_56_NaO_27_]^+^; 907.3	[C_53_H_78_NaO_31_]^+^; 1233.4	[C_18_H_30_NaO_15_]^+^; 509.1	[C_24_H_42_NaO_21_]^+^; 689.2
		[C_36_H_60_NaO_30_]^+^; 995.3	[C_24_H_40_NaO_20_]^+^; 671.2	[C_32_H_44_NaO_14_]^+^; 675.3	
[C-9 + Na]^+^ MS/MS	R_1_ = Hex_7_; R_2_ = Hex_2_	[C_74_H_114_NaO_49_]^+^; 1809.65			
		[C_62_H_94_NaO_39_]^+^; 1485.5	[C_62_H_93_NaO_38_]^+^; 1468.5	[C_42_H_69_NaO_35_]^+^; 1156.3	[C_56_H_84_NaO_34_]^+^; 1323.5
		[C_36_H_60_NaO_30_]^+^; 995.3	[C_30_H_52_NaO_26_]^+^; 851.3		

**Fig. 2:**
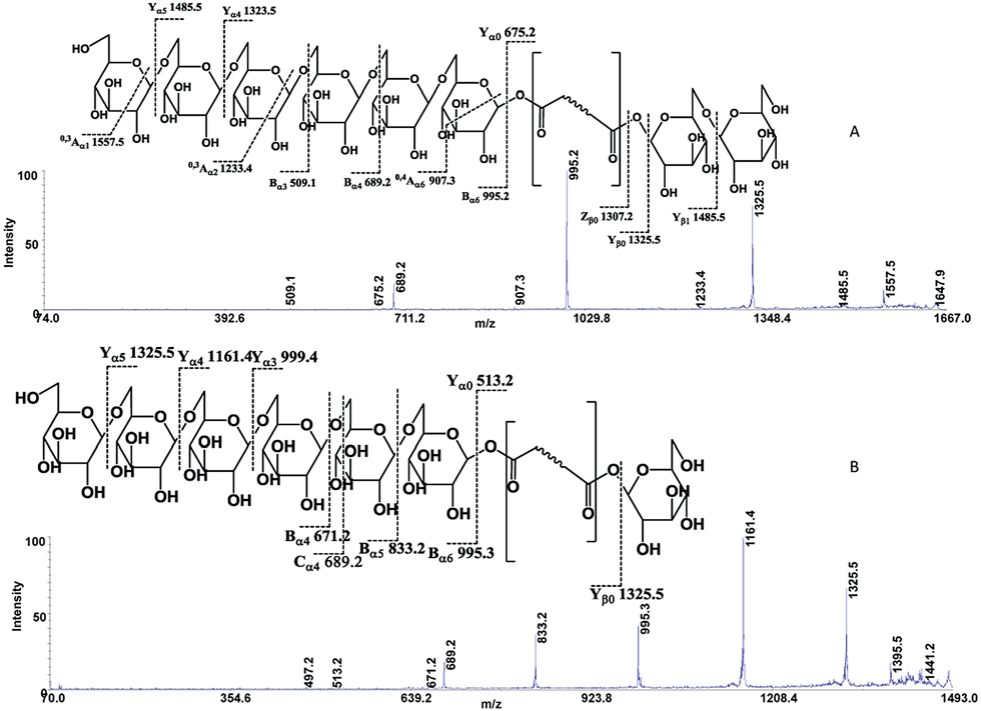
High-energy CID-MS/MS spectra of the (A) [M + Na]^+^ ion of *m*/*z* 1647.59 for C-8; (B) [M + Na]^+^ ion of *m*/*z* 1485.54 for C-7.

The following three groups of product ions were observed: (1) B_α_- and Y_α_-type (B_β_- and Y_β_) ions that allowed us to determine the glycosyl composition of individual crocins; (2) charge-remote fragmentations of glycosidic substituents; (3) charge-remote cross-ring fragmentations (A-type ions). On the basis of high energy CID-MS/MS data, the precursor ions of *m*/*z* 513.21, 675.27, 837.32, and 999.38 were ascribed to C-1–C-4 sodium adducts (Table 1). The glycosyl compositions (Hex)_1_Ct, (Hex)_2_Ct, (Hex)_2_Ct(Hex)_1_, and (Hex)_2_Ct(Hex)_2_ were assigned to crocins C-1–C-4, respectively. A detailed investigation of the product ions was performed by product ion scan of the C-5 ([M + Na]^+^, *m*/*z* 1161.45; Table 1) in order to establish the presence of isomeric structures. The group of the product ions B_α2_ (*m*/*z* 347.1), B_α3_ (*m*/*z* 509.1), Z_β2_ (*m*/*z* 837.3), Y_β2_ (*m*/*z* 999.4), Y_α1_ (*m*/*z* 675.3) and B_β2_ (*m*/*z* 347.1), corroborated the isomeric structure (Hex)_3_Ct(Hex)_2_. Nevertheless, the product ions of *m*/*z* 671.2 ([C_24_H_40_NaO_20_]^+^), 513.2 ([C_26_H_34_NaO_9_]^+^), 819.3 ([C_38_H_52_NaO_18_]^+^), and 689.2 ([C_24_H_42_NaO_21_]^+^) were diagnostic for a tetraglycosyl chain. These data indicated that the ion of *m*/*z* 1161.45 arose from an isomeric mixture of the pentaglycosylated crocetins (Hex)_3_Ct(Hex)_2_ and (Hex)_4_Ct(Hex)_1_. Furthermore, the absence of ion signals due to the ^0,4^A_2_ and the ^3,5^A_2_ cleavages clearly indicated linear glycosidic chains. The elucidation of unknown crocins C-8 and C-7 is illustrated in Fig. 2A–B. Based on the accurate mass analysis, the quasimolecular ion [M + Na]^+^ (*m*/*z* 1647.59) corresponded to a new crocin bearing eight glucose residues Ct(Hex)_8_ (Table 1, Fig. 2A). The high energy CID-MS/MS fragmentation of neutral oligosaccharides often occurs from the non-reducing end toward the reducing end. Therefore, the consecutive losses of Hex from the quasimolecular ion, produced ions of *m*/*z* 1485.5 and *m*/*z* 1325.5, respectively, indicating that Hex glycosyl residues were on the non-reducing ends of the crocin C-8. Accordingly, Ct(Hex)_8_ was recognized as a bis-substituted glycosyl esters of crocetin. The complementary product ions of *m*/*z* 675.2 (Y_α0_) and 995.2 (B_α6_) suggested that one of the glycosidic chains is composed by six glucose residues. Furthermore, the ions sequence B_α3_, B_α4_ and B_α6_ were sufficient to determine the sequence of the carbohydrate moiety and to confirm the glycosyl composition as (Hex)_6_Ct(Hex)_2_ for crocin C-8, as shown in the inset structure (Fig. 2A). The high energy CID-MS/MS spectrum of the unknown crocin C-7 ([M + Na]^+^ at *m*/*z* 1485.54; Table 1) is shown in Fig. 2B. The losses of two Hex residues from the quasimolecular ion gave the product ions of *m*/*z* 1325.5 and 1161.4, indicating that this glycosyl unit was present at both the non-reducing ends. The highest B type product ion of *m*/*z* 995.3 was assigned as [6Hex + Na]^+^. Accordingly, the crocin C-7 contains a hexaglycosyl chain as substituent. Again, the ion sequence of the product ions B_α4_, B_α5_ and B_α6_ indicated a linear structure for the hexaglycosyl chain. Furthermore, the formation of the product ions Y_α0_ (*m*/*z* 513.2) and Z_α0_ (*m*/*z* 497.2) retaining the aglycon moiety, confirmed the composition as (Hex)_6_Ct(Hex)_1_ for the precursor C-7. Similarly, the structure (Hex)_4_Ct(Hex)_2_ and (Hex)_7_Ct(Hex)_2_ were assigned to crocins C-6 and C-9, respectively. Table 1 summarizes the observed MS and MS/MS signals and the composition of all crocins C-1–C-9. The complexity of the MALDI-MS spectrum indicated the large presence of low molecular weight *m*/*z* signals assigned to various metabolites. The use of the acid solution^28^ led to the extraction of a chemically homogeneous class of compounds. Several glycoconjugated carotenoid breakdown products showing a similar “trimethylcyclohexene” skeleton were hypothesized to be present in stigmas of *Crocus sativus* L.^29^ picrocrocin ([4-(β-d-glucopyranosyl)-2,6,6-trimethyl-1-cyclohexene-1-carboxaldehyde]) is the major secondary metabolites of saffron.^1,2,5,30^ Since picrocrocin have been shown to exhibit specific fragmentation patterns in ESI-MS/MS,^5^ we hypothesized that high energy MS/MS analysis could support the identification of known or unknown carotenoid-derived metabolites in saffron. The main target of MS/MS experiments was to identify compounds belonging to a certain class of metabolites. The resulting spectra were compiled and manually evaluated. This search allowed us to compile different groups of high energy MS/MS spectra. The first group contained spectra displaying the ability of metabolites to lose 1 or 2 Hex residues (162, 324 Da) resulting in the aglycone ion (Y_0_) of *m*/*z* 369 and 339 complemented by fragment ions of *m*/*z* 207, 191, and 177. The second group comprised spectra showing the ability of metabolites to lose 1 or 2 Hex residues (162 and 324 Da, respectively; Y-ions); MS/MS daughter ions corresponding to [M + H-Hex]^+^ (Y_0_), and [M + H-120]^+^ (^0,2^X) were considered as characteristic fragments for the identification of *O-*glycosides. These considerations led us to recognize the first group as composed by glycoconjugated of “trimethylcyclohexene” (TMC) skeleton, Hex-*O*-C type structures; while glycosylated metabolites, such as flavonoids, were collected in the second group.

### Identification of carotenoid-derived metabolites

The cation adduct of the picrocrocin [M + K]^+^ was detected at *m*/*z* 369.13 ([C_16_H_26_O_7_K]^+^, Δppm = 6.2, DBE = 3.5). High energy CID-MS/MS (1 kV) induced extensive cross rings fragmentation providing valuable information for the characterization either the basic “trimethylcyclohexene” (TMC) skeleton and the sugar moiety. In particular, the cleavage at the glycosidic *O*-linkages with a concomitant H-rearrangement led to the elimination of 162 Da (hexose) yielding the Y_0_ ion of *m*/*z* 207.1, base peak of the spectrum. However, the most useful fragmentations in terms of TMCs identification are those that require cleavage of two C–C bonds of the C-ring, resulting in structurally informative ^i,j^TMC ions. The ions of *m*/*z* 167.1, 149.1 and 109.1 were due to the done by ^3,4^TMC^+^, ^3,5^TMC^+^ and ^3,6^TMC^+^ cleavages and indicated two, and one CH_3_ group at C6 and C2, respectively. In addition, losses of small molecules and/or radicals from the [M + K]^+^ ion was observed. Losses of 15 Da (CH_3_), 18 Da (H_2_O), 30 Da (CH_2_O), and the successive loss of these small groups were detected.

**Fig. 3:**
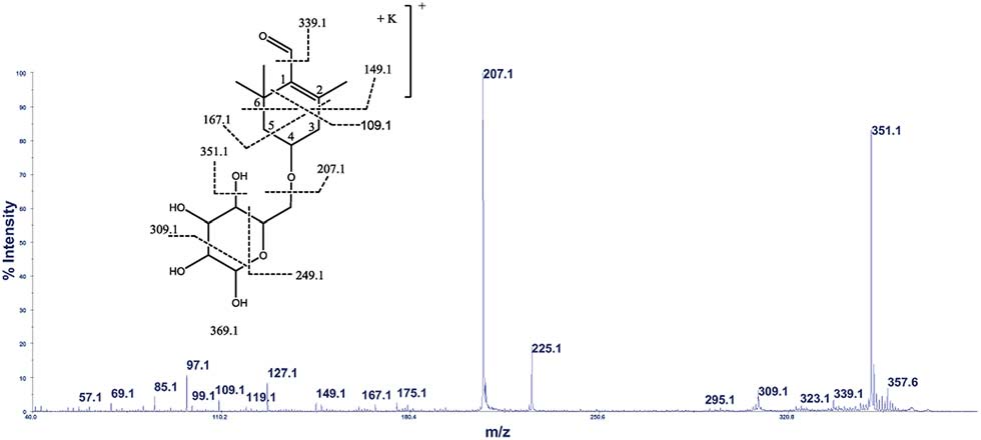
High-energy CID-MS/MS spectra of the [M + K]^+^ ion of *m*/*z* 369.13.

**Scheme 1:**
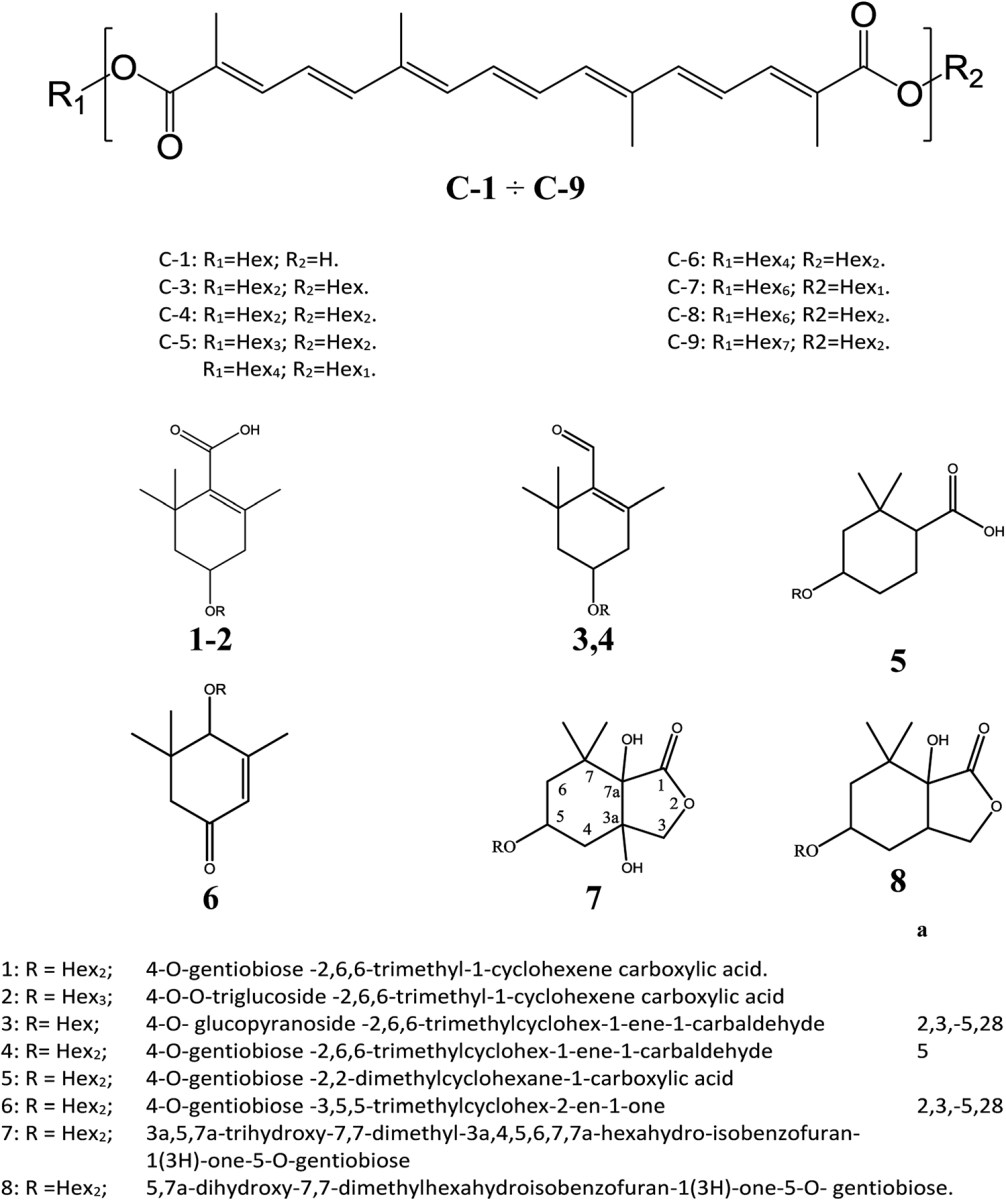
Crocins and carotenoid-derived metabolites identified by MALDI MS and MS/MS.

**Fig. 4:**
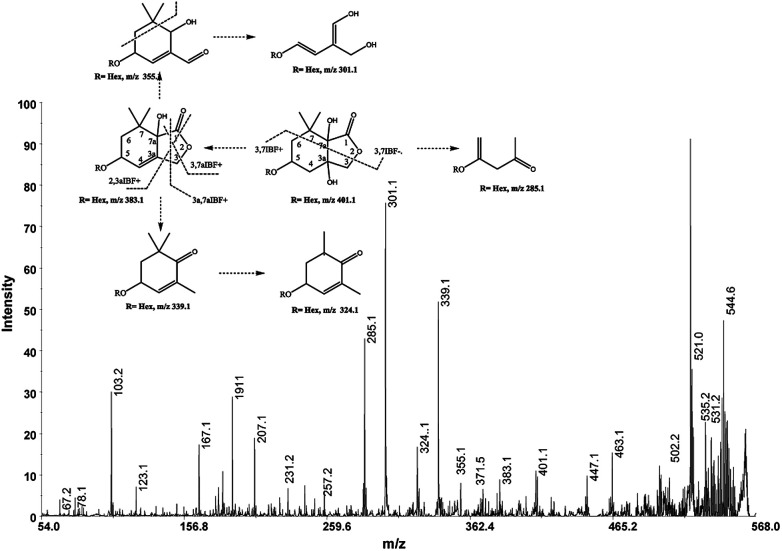
High-energy CID-MS/MS spectra of the [M + Na]^+^ ion of *m*/*z* 563. 19.

These mass losses were helpful to confirm the presence of specific functional groups in the structure. Thus, the CHO group at C1 was detected by the loss of 30 Da (CH_2_O, *m*/*z* 309.1) from the [M + K]^+^ precursor ion (Fig. 3). The MALDI-TOF-MS signals at *m*/*z* 531.20 ([C_22_H_36_O_13_Na]^+^, 1), 693.26 ([C_31_H_38_NaO_16_]^+^, 2), *m*/*z* 353.16 ([C_16_H_26_NaO_7_]^+^, 3), 515.20 ([C_22_H_36_NaO_12_]^+^, 4), 519.21 ([C_21_H_36_NaO_13_]^+^, 5), 501.19 ([C_21_H_34_NaO_12_]^+^, 6), 563.19 ([C_22_H_36_O_15_Na]^+^, 7) and 547.20 ([C_22_H_36_NaO_14_]^+^, 8) were assigned to compounds 1–8, respectively (Scheme 1). The compounds were identified on the basis of the accurate mass of each MALDI-TOF-MS signal and the high energy CID-MS/MS fragmentation behaviors. For instance, the high energy CID-MS/MS spectrum of the molecular ion [M + H]^+^ (*m*/*z* 531.20, [C_22_H_36_NaO_13_]^+^, Δppm = 5.2, DBE = 4.5) showed the diagnostic product ions of *m*/*z* 369.1, and 207.1 together with a radical product ion [C_10_H_16_O_3_]^+^ of *m*/*z* 184.1. The last two species of *m*/*z* 207 and 184 arose from two competitive fragmentation pathways, the heterolytic and the homolytic^31^ cleavage of the glycosidic bond, respectively. The detected ^3,4^TMC^+^ (*m*/*z* 167.1), and ^3,5^TMC^+^ (149.1) ions confirmed the presence of CH_3_ groups at C2 and C6. The elucidation of unknown 7 is illustrated in Fig. 3. High energy CID-MS/MS of the precursor ion [7+H]^+^ (*m*/*z* 563.19, Δppm = 6.0, DBE = 5.50, [C_22_H_36_O_15_Na]^+^) showed species arising from the direct and consecutive loss of small molecules allowing the complete characterization of the hexahydro-isobenzofuran-1(3*H*)-one (IBF) moiety (Fig. 4). A glycoconjugated showing an IBF structure was already observed.^5^ The loss of 18, 28, and 44 Da from the parent ion, species of *m*/*z* 545.1, 535.2, 521.0 respectively, indicated that OH and CH_2_O_2_ groups were located on the aglycon. The formation of Y_1_ series of ion differing each other of 18 (401 → 383), 28 (383 → 355), 44 (383 → 339) suggested the presence of the fused cyclic lactone. Fragments ^2,3a^IBF^+^ (207.1), ^3a,7a^IBF^+^ (181.1), ^2,3a^IBF^+^-15 (191.1), ^3,7a^IBF^+^ (151.1) and ^2,7^IBF^+^ (123.1) allowed to confirm the fused cyclic lactone structure and the presence of methyl substituents at C7. High energy CID-MS/MS of the precursor ion [8 + Na]^+^ (*m*/*z* 547.20) yielded Y and IBF ion types by cross ring fragmentations of the heterocycle. The product ions of *m*/*z* 385.1, 355.1, 325.1 and 301.1 were characteristic Y_1_ fragments generated by glycosidic bond cleavage and direct and consecutive neutral losses of CO (385 → 355), HCOH (355 → 323) and C_4_H_8_ (355 → 301) from the IBF skeleton. Characteristic fragments of the heterocycle were observed at *m*/*z* 123.1 (^2,7^IBF^+^), 191.1 (^2,3a^IBF^+^), 165.1 (^3a,7a^IBF^+^) and 135.1 (^3,7a^IBF^+^).

### Identification of flavonols

Flavonol compounds, such as kaempferol-3-sophoroside, kaempferol-3-sophoroside-7-glucoside, kaempferol tetrahexoside and kaempferol-3-dihexoside have already been described in *Crocus sativus* L.^30^ The MALDI-TOF-MS spectrum of saffron extract displayed ion signals at *m*/*z* 773.21 ([M + H]^+^, [C_33_H_41_O_21_]^+^; Δppm = 6) and 633.15 ([M + Na]^+^, [C_27_H_30_NaO_16_]^+^; Δppm = 7), respectively. CID MS/MS spectrum of precursor ions [M + H]^+^ at *m*/*z* 773.21 and 633.15 allowed us to identify 3-sophoroside-7-glucoside and kaempferol-3-sophoroside, respectively (Fig. 2S†). In addition, the detected ions [M + H]^+^ of *m*/*z* 303.05 ([C_15_H_11_O_7_]^+^, Δppm = 7) and 465.10 ([C_21_H_21_O_12_]^+^, Δppm = 6) were ascribed to quercetin and quercetin 3-*O*-glucoside, respectively. The structural assignment was performed on the basis of the high energy CID-MS/MS fragment ions (Fig. 3S†).

### MALDI MS analysis of adulterant and authentic adulterated saffron samples

**Fig. 5:**
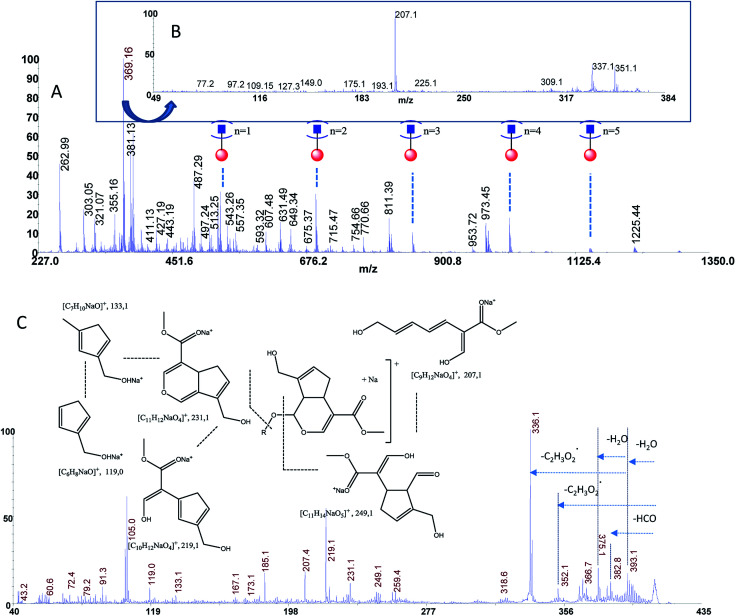
Panel (A) MALDI MS spectrum of sample S3, Panel (B) MS/MS spectrum of the ion of *m*/*z* 369.13 from sample S3, Panel (C) MS/MS spectrum of geniposide from sample S3.

Saffron authentication aims to identify specific changes between control samples (genuine) and the adulterated one, focusing on a particular subset of metabolites (crocins and picrocrocin). The challenge is the simultaneous detection of species arising from saffron and adulterant with sufficient abundance for quantitative and MS/MS analysis, minimizing sample manipulation. Thus, chemical component profile of authentic saffron samples adulterated with vegetable matter (*Curcuma longa*, *Calendula officinalis* L.) were evaluate by MS and MS/MS. MS/MS induced cleavages of glycosidic bonds ([M-Hex]^+^; [M-2Hex]^+^) led to product ions containing the aglycone molecule and the aglycone itself. Thus, the ion species of *m*/*z* 641.17 ([C_28_H_33_O_17_]^+^), 641 → 478 → 339), *m*/*z* 649.14 ([C_27_H_30_NaO_17_]^+^, 649 → 487 → 325), and *m*/*z* 617.15 ([C_27_H_30_NaO_15_]^+^, 617 → 455 → 309) detected in the sample adulterated with calendula were attributed to isorhamnetin-, quercetin- and kaempferol-di-*O*-glycoside, respectively. Accordingly to the literature, the identified isorhamentin glycoside represents a specific biomarker of calendula.^9,30^ Representative MALDI spectra of saffron, adulterated sample (2%), and turmeric are shown in Fig. 4S.† The presence of a little amounts of turmeric (lower than 1–2%, w/w) was sufficient to produce sensitive effects on spectral data. The low mass region of the spectrum displayed ions arising from the major components of turmeric. The detected ion species of *m*/*z* 369.13 ([C_21_H_21_O_6_]^+^), 339.12 ([C_20_H_19_O_5_]^+^) and 309.11 ([C_19_H_17_O_4_]^+^) were assigned to curcumin, demethoxycurcumin and bisdemethoxycurcumin, which are the predominant curcuminoids in turmeric.^32^ The fragmentation behavior of the major curcuminoids was extensively studied and the main fragmentation channels of these compounds were elucidated.^33^ Having identified crocin, picrocin and some carotenoid-derived metabolites by MALDI MS and MS/MS from authentic saffron samples (Scheme 1), MALDI MS/MS experiments were performed to evaluate the overlap between specific ion signals of saffron, calendula and turmeric. Unexpectedly, curcumin ([C_21_H_21_O_6_]^+^, theoretical mass 369.1333) and the cation adduct of picrocrocin ([C_16_H_26_O_8_K]^+^, theoretical mass 369.1310 showed to be isobars with Δ*m*/*z* < 0.0023, thus not resolvable by TOF. On the contrary, direct MS/MS analysis of the ion of *m*/*z* 369.13 enabled the rapid and unambiguous distinction between curcumin and picrocrocin whose structures were univocally confirmed (Fig. 5S†). Since interference was observed for only one endogenous molecule (picrocrocin *m*/*z* 369.13) during the analysis of authentic adulterated samples, the developed approach was thought to be useful to determine saffron adulteration regardless of the type of adulterant.

In absence of any chromatographic step, quantitation can be performed using non-isotopic isobaric internal standard. The endogenous metabolite and the isobaric compound are measured by MALDI MS. Since the amount of internal standard is known, and the amounts of internal standard and analyte can be determined from the mass spectra, the amount of the endogenous analyte can be calculated. These consideration leads to use curcumin as non-isotopic isobaric internal standard for saffron authentication.

### Calibration curve experiments

A synthetic set of 8 adulterated authentic saffron samples with curcumin in the range of 0–30% was prepared. Thus, eight sets of synthetic sets of adulterated authentic saffron sample spots were processed through a simple “sample list”. MS analysis were performed in triplicate using three spots for each sample. The linear range was assessed by plotting the mole ratio of the analyte (MR_s_) *vs.* the percentage of adulteration with curcumin. Molar ratio was evaluated by the equation MR_S_ = ∑ICA_S_/(∑ICA_S_ + ∑ICA_T_), where ICA_S_ and ICA_T_ are the isotope cluster areas of specific ion signals from saffron and curcumin, respectively. In all set only C-2–C-4 and curcumin ion signals were used for data analysis. The calibration curve built using triplicate of synthetic sets of adulterated authentic saffron samples showed good linearity (*R*^2^ = 0.996), and with confidence intervals at 95% for intercept including the zero value. The ANOVA regression model was significant, *F*(1, 72) = 16 440.31, *p* < 0.001, *y* = (0,0120 ± 0.0001)*x* + (−0,1482 ± 0.0081) (Fig. 6S†). The maximum adulteration percentage was estimated (98.9%) interpolating the limit of detection (LOD) in linear regression. LOD and LOQ were 1.1% and 6% respectively. The repeatability calculated on spiked samples was found to be lower than 1% (RSD%). Accuracy of the method was determined by using fortified samples prepared by adding known quantities of the adulterant (citrus leaves, calendula) to authentic saffron samples (Table 2).

**Table 2:** Analytical parameters

	a%	b%	*RSD%	Accuracy %
SPIKED 1	12.60	12.35 ± 0.26	2.13	98.0
SPIKED 2	9.86	9.53 ± 0.22	2.31	97.0
SPIKED 3	25.00	24.45 ± 0.31	1.28	98.0

a % (w/w) of adulteration.

b % of adulteration calculated. *the reproducibility of the measurements was determined by extracting the same sample in triplicate over a period of 1 week.

Quantitative recovery and high reproducibility (*RSD%) highlighted the reliability of the method, suggesting that the developed approach is suitable for a rapid screening of saffron. Comparison between the determined LOD with values obtained as previously reported employing HPLC/PDA and/or ESI-MS detection, namely 5% (w/w) for calendula or safflower and 2% (w/w) for turmeric,^9^ implied that the proposed approach may enable detection at lower levels of plant-derived adulterants in saffron. The recently published and highly sensitive methods that combine LC and MS to assess the authenticity of saffron through the analysis of a group of kaempferol derivatives^11^ and through geniposide^12^ have LOD ranging from 0.2–2% and 10 ng mL^−1^ respectively. However, the accuracy of the most sensitive method (LOD 10 ng mL^−1^), assessed evaluating the recovery obtained for geniposide in spiked saffron sample with 1 μg mL^−1^ of geniposide, was 89 ± 14%.^12^ The relatively poor temporal resolution, due to sampling times of up to 20 minutes, is the major limit. Throughput of the LC MS systems is restricted to a limited number of samples. The MALDI MS method here described shows LOD comparable to that of LC/MS methods. However, in our case, chromatographic separations or desalting are not required, and the recovery is quantitative. Moreover, the MALDI-MS method enables a high sample throughput because of the minimal sample preparation and the very short measuring time per sample. Since no interference was observed for both endogenous molecule and the non-isotopic isobaric internal standard during the analysis of authentic and spiked samples, the developed approach could be used to determine saffron adulteration regardless of adulterant type. Furthermore, the reliability of the proposed approach was confirmed by analytical parameters of accuracy calculated using spiked samples prepared by adding known quantities of citrus leaves and calendula (Table 2). Spiked samples given accuracy higher than 97% (Table 2). Recovery of the analyte was quantitative because no chromatographic step is involved in sample preparation.

The good performance of the method was shown also by the repeatability of the measurements, with the *RSD% value being lower than 2%, in all the examined cases (Table 2). The method was further applied to saffron sachets which were available at local stores (Table 3). These samples were chosen on the basis of their low cost and questionable origin.

**Table 3:** Adulteration percentage calculated and precision as obtained for the measurements carried out on suspicious saffron sample

	%	RSD%
S1	10.47 ± 0.13	1.25
S2	25.25 ± 0.43	1.72
S3	21.07 ± 0.41	2.00

### MALDI MS analysis of suspicious saffron samples (S1–S3)

The estimated adulteration percentage for saffron samples (S1–S3) ranged from 10% to 25% (Table 3). MALDI MS spectra of these samples (S1–S3) showed similar molecular profile. Representative MALDI spectrum of suspicious saffron samples (S3) are shown in Fig. 5A. MS/MS spectra of the ion specie of *m*/*z* 369.13 were acquired in all case in order to establish the presence of turmeric as adulterant (Fig. 5B, sample S3). The formation of the daughter ions of *m*/*z* 351, 337, 309, 225, 207 indicated the presence of picrocrocin and able us to exclude the presence of turmeric as adulterant. Analogously, MS/MS of the signal of *m*/*z* 641 able us to exclude the presence of calendula as adulterant. Considering that gardenia could be used as adulterant in S1–S3, and that it is very difficult to detect using classical methods because it shares with saffron several secondary metabolites, crocins C1–C3 and flavonoids, MS/MS experiments were performed in order to detect geniposide in crude extracts. According to MS, MS/MS experiments, and the studies previously reported, the ions of *m*/*z* 411.13 ([C_17_H_24_O_10_Na]^+^, Δppm = 5) and 427.00 ([C_17_H_24_O_10_K]^+^, Δppm = 5) were assigned to sodium and potassium adducts of geniposide, respectively (Fig. 5C). Geniposide, is a sensitive biomarker for the detection of adulteration of saffron samples, since represents the main component of extract of fruit gardenia.^12^ The detection of geniposide in all samples S1–S3 indicated adulteration by *Gardenia jasminoides*.

## Conclusions

MALDI MS of saffron extracts, which has the advantage of focusing on the more polar components, was found to be a fast and selective technique to control quality and to evaluate adulteration level. No matrix effects were observed and good results were obtained with respect to instrumental repeatability (*RSD% < 2%) and LOD (1.1%). The analysis of commercial samples of saffron using the proposed approach showed the suitability of the method in routine analysis (minimal sample preparation and very short measuring time per sample). The data here reported showed that the proposed approach enables quantitation of adulteration percentage in saffron, regardless of adulterant identity, and identification of the adulterant itself.

## Conflicts of interest

There are no conflicts to declare.

## Supplementary Material

RA-008-C8RA07484D-s001
