# Supplementary material for: Molecular species fingerprinting and quantitative analysis of saffron (Crocus sativus L.) for quality control by MALDI mass spectrometry
Source: RSC Adv. 2018 Oct 23;8(63):36104–13. doi: 10.1039/c8ra07484d (PMC9088749; doi:10.1039/c8ra07484d)
Supplement: RA-008-C8RA07484D-s001 [file RA-008-C8RA07484D-s001.pdf]

Electronic Supplementary Material (ESI) for RSC Advances.
This journal is © The Royal Society of Chemistry 2018

Molecular species fingerprinting and quantitative analysis of saffron (*Crocus sativus* L.) for quality control by MALDI mass spectrometry.

Donatella Aiello[a], Carlo Siciliano*[b], Fabio Mazzotti[a], Leonardo Di Donna[a], Constantinos M. Athanassopoulos[c], Anna Napoli*[a]

[a.] *Department of Chemistry and Chemical Technologies, University of Calabria, Italy.*
[b.] *Department of Pharmacy, Health and Nutritional Sciences, University of Calabria, Italy.*
[c.] *Department of Chemistry, University of Patras, Patras, Greece.*

Figure 1S. MALDI MS spectrum of saffron using (A) C_2H_5OH,100% (B) CH_3OH/H_2O: 50/50 (v/v), (C) CH_3OH/NH_4HCO_3:50/50 (v/v). ★ MALDI matrix

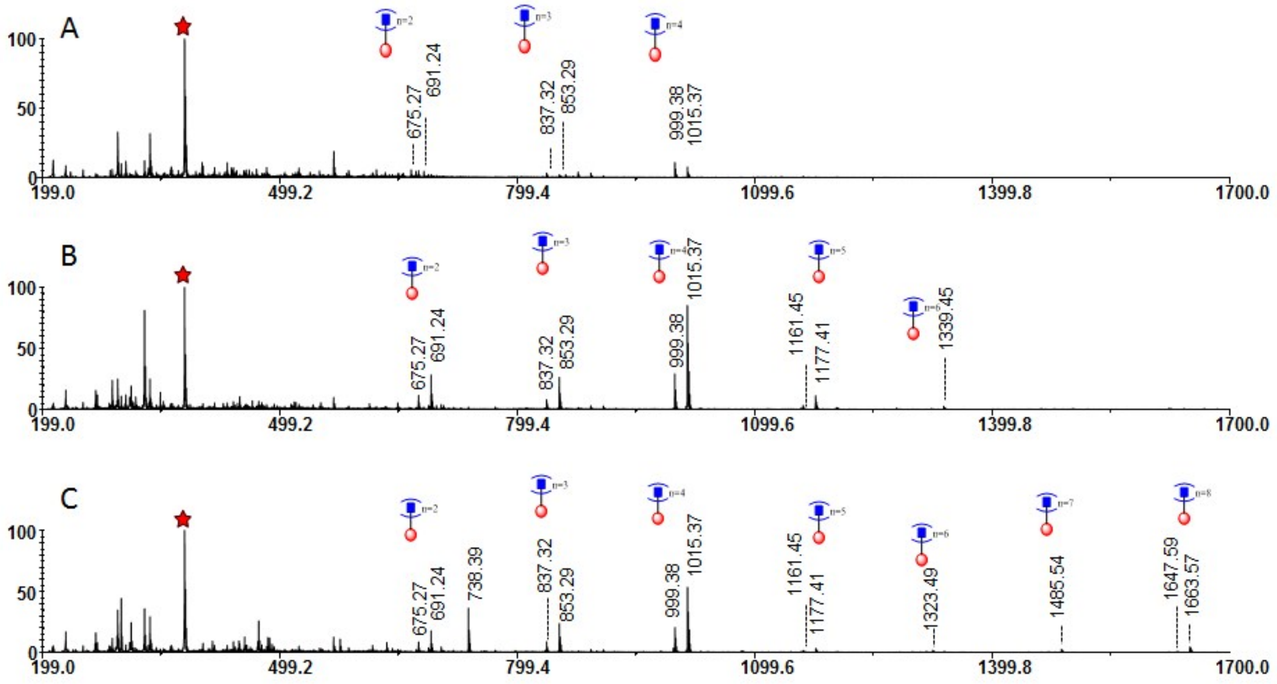

Figure 2S. MS/MS spectrum of (A) Kaempferol 3-sophoroside-7-glucoside ($[C_{33}H_{41}O_{21}]^+$; 773.21, Δppm = 6); (B) Kaempferol 3-sophoroside ($[C_{27}H_{30}NaO_{16}]^+$; 633.15, Δppm = 7)

A

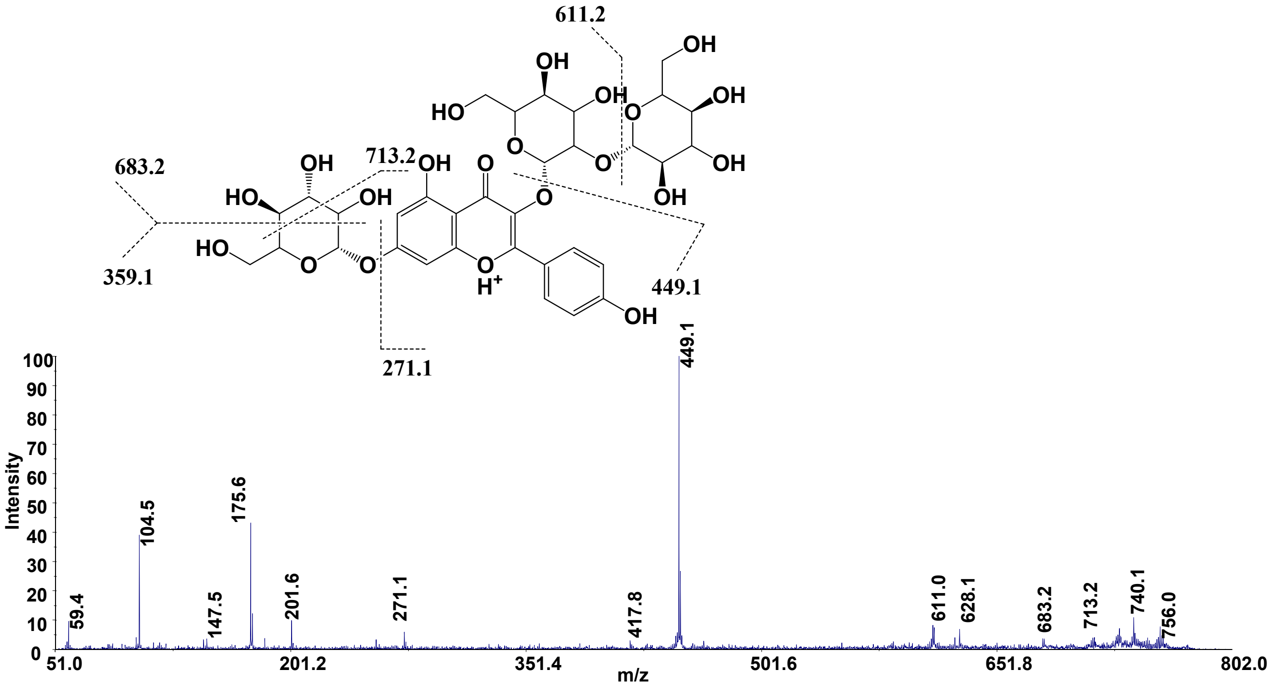

B

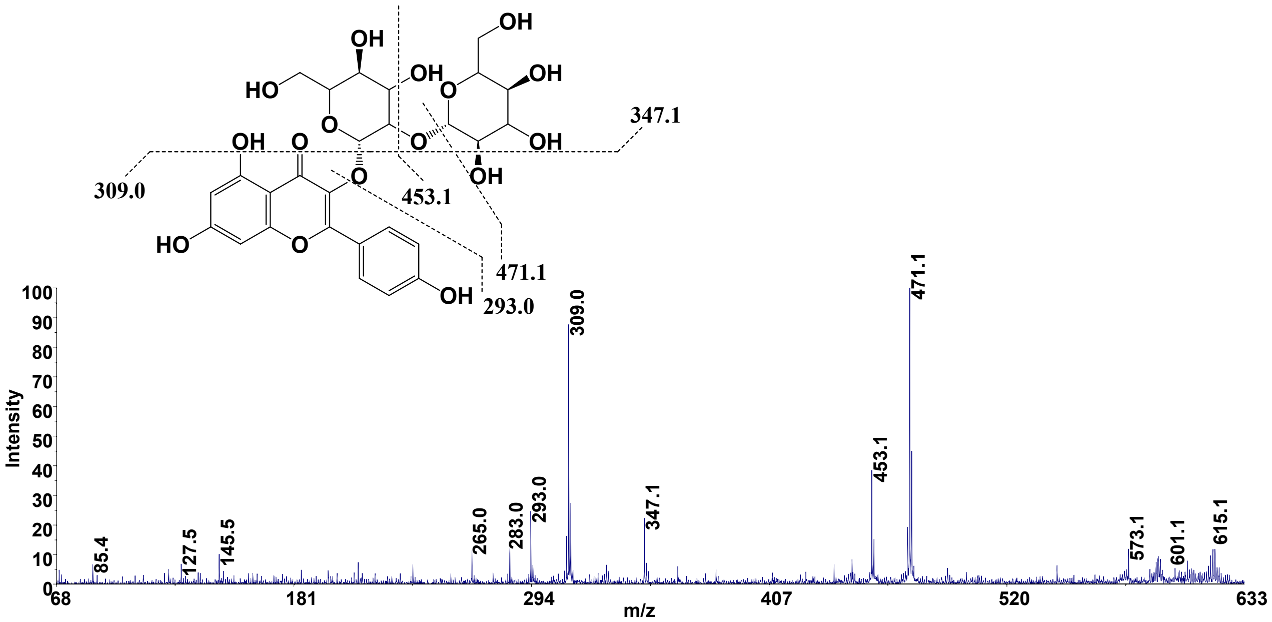

Figure 3S. MS/MS spectrum of (A) quercetin (m/z 303.05 ; ppm = 7), (B) quercetin 3-*O*-glucoside (m/z 465.10 ppm = 6)

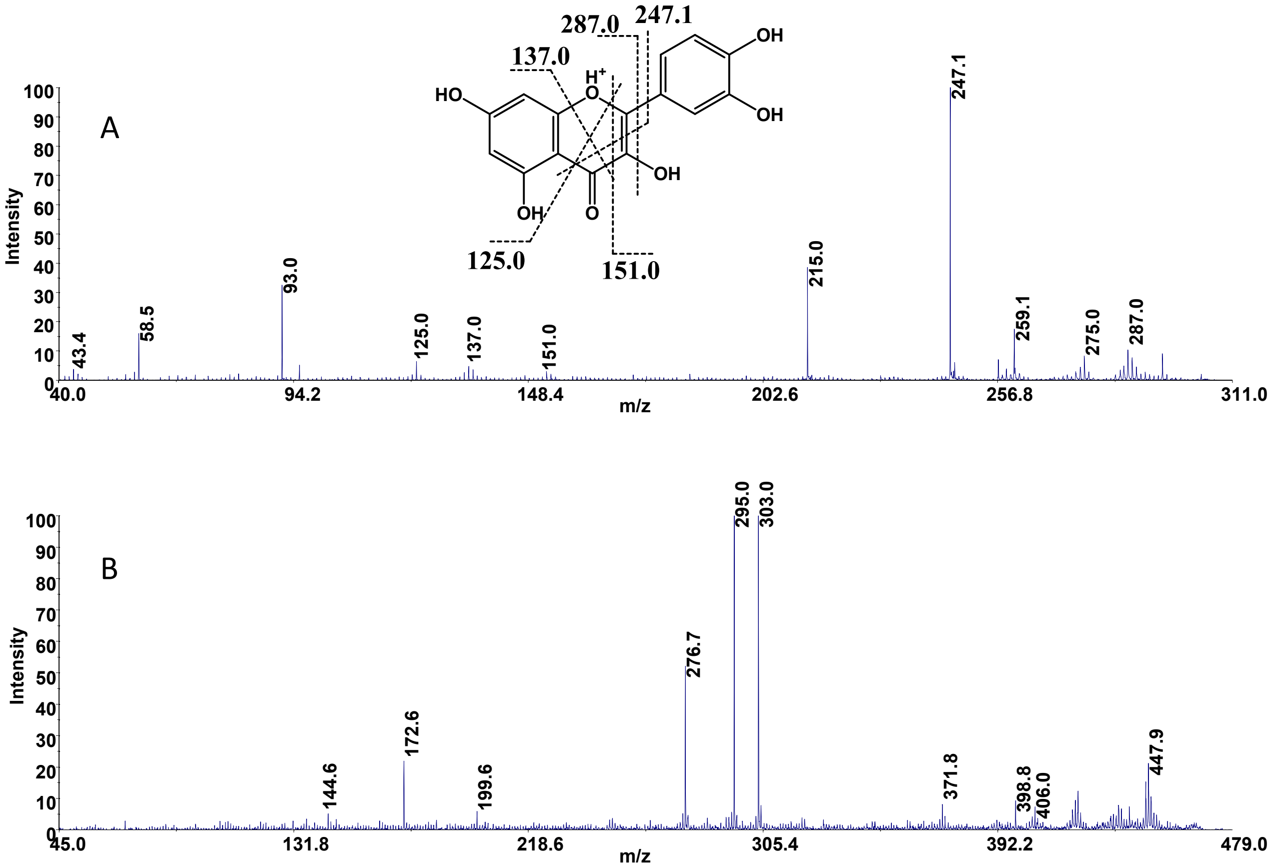

Figure 4S. MALDI spectra of (A) authentic saffron, (B) adulterated sample with turmeric (2%) and (C) turmeric.

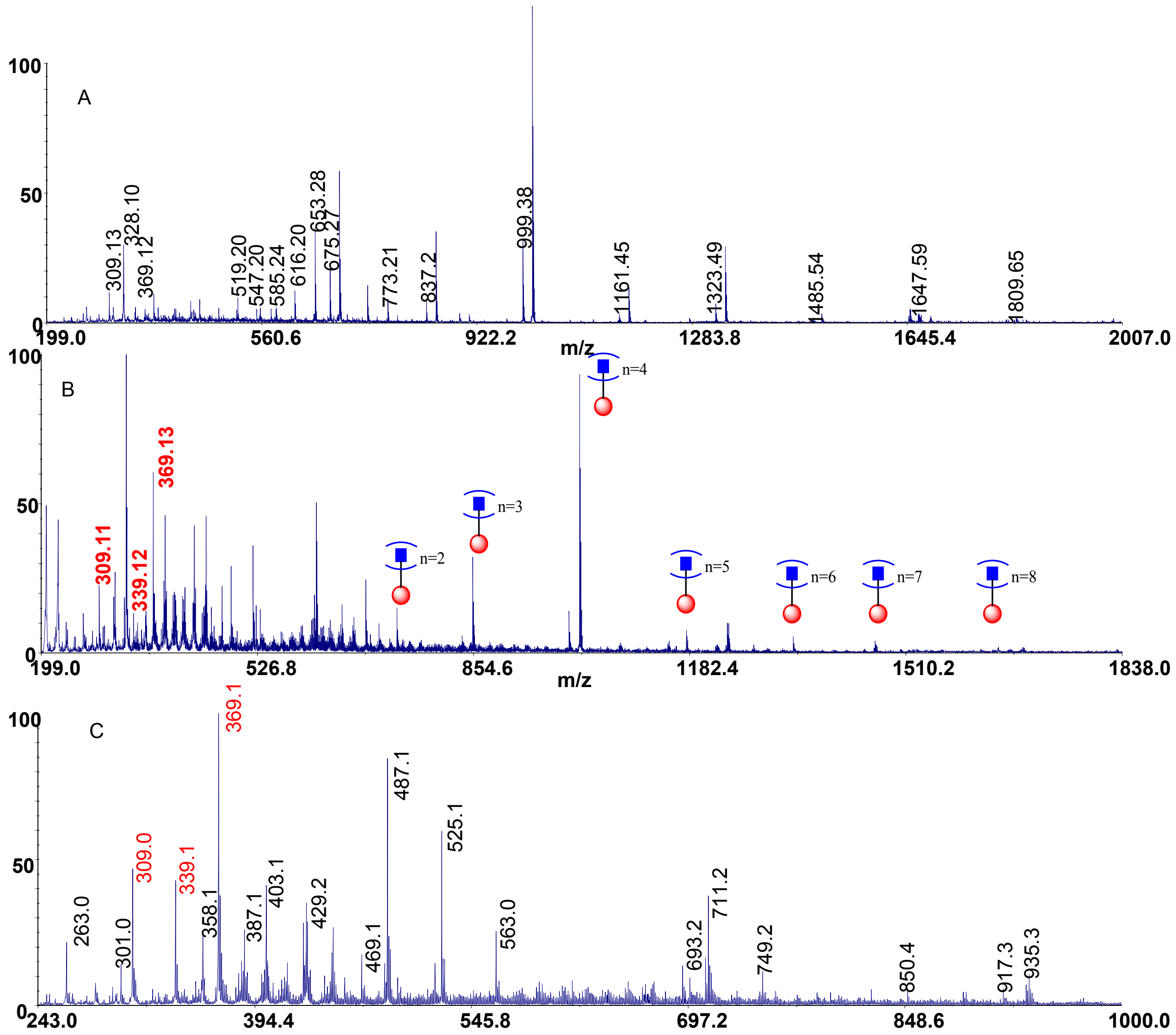

Figure 5S. MALDI MS/MS spectra of (A) picrocrocin and (B) curcumin.

A

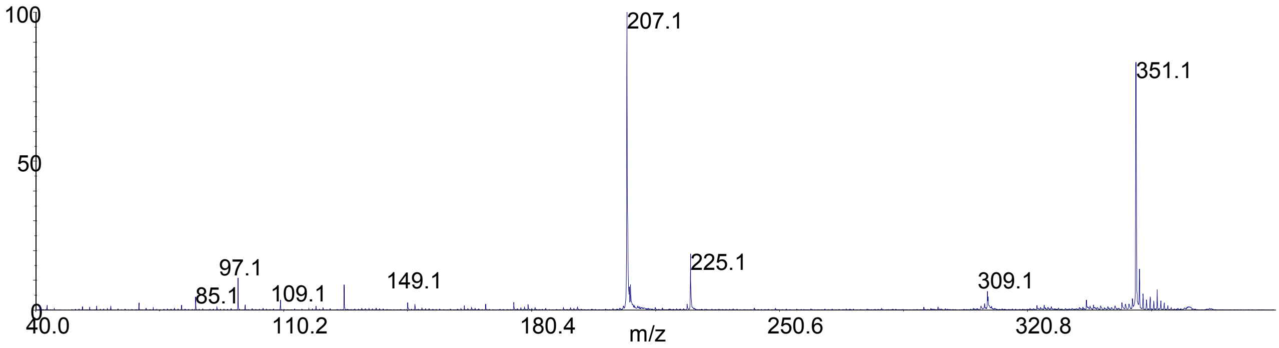

B

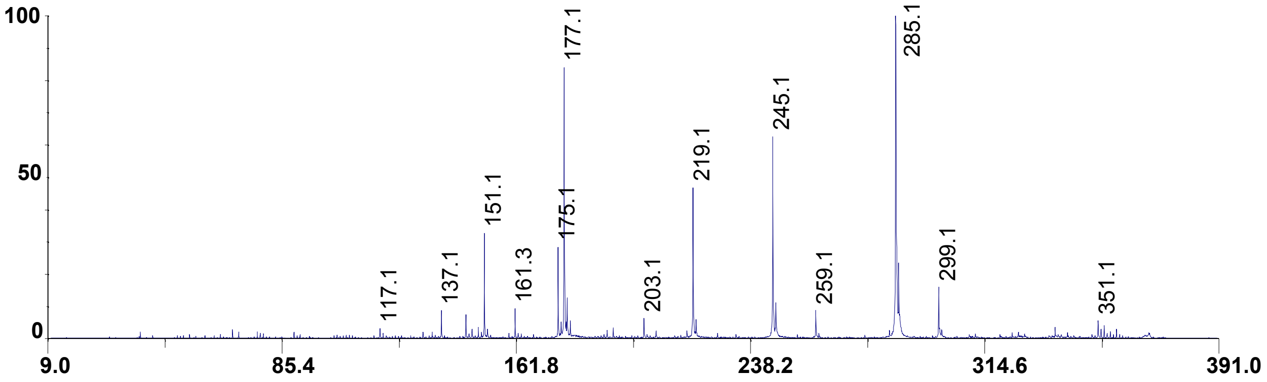

Figure 6S. Working calibration curve.

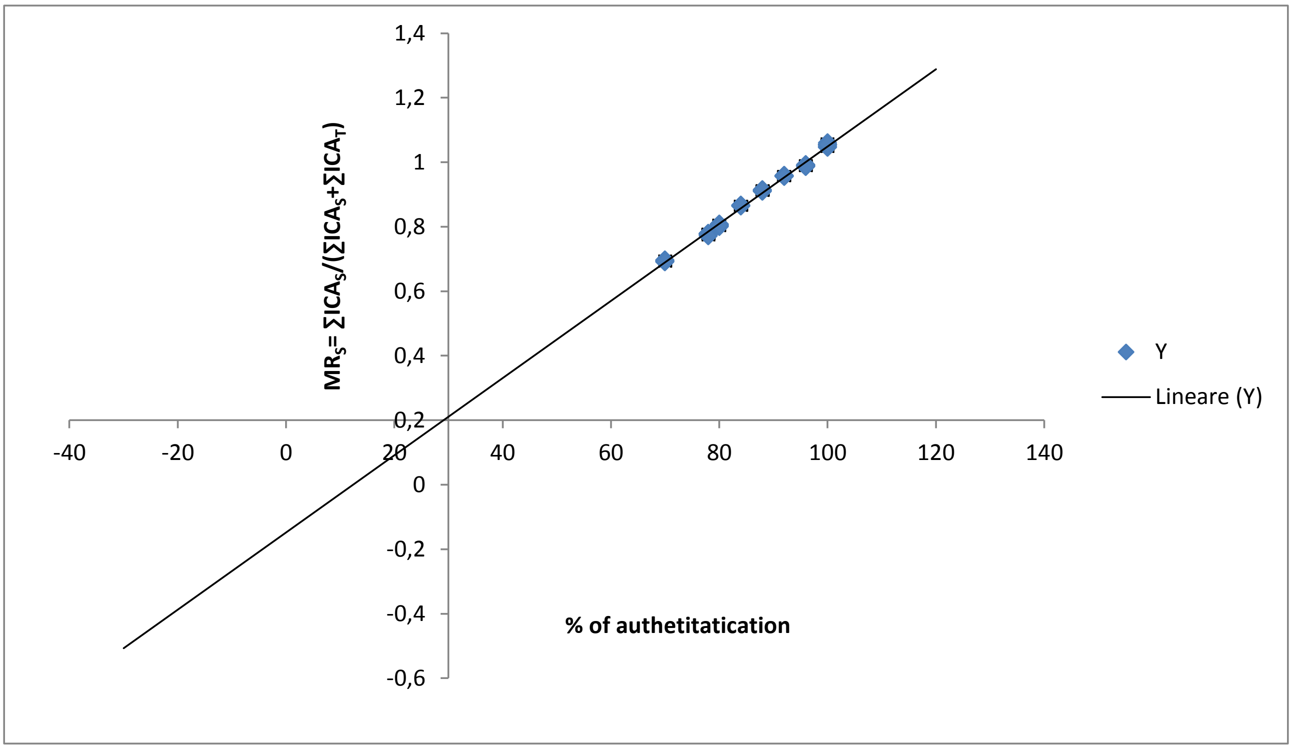

Intercept	-0,1482 ± 0,0081
Slope	0,0120 ± 0,0001
